# BRD4 regulates self‐renewal ability and tumorigenicity of glioma‐initiating cells by enrichment in the Notch1 promoter region

**DOI:** 10.1002/ctm2.181

**Published:** 2020-10-04

**Authors:** Zhennan Tao, Xuetao Li, Hao Wang, Guangliang Chen, Zibin Feng, Yue Wu, Haoran Yin, Guozheng Zhao, Zhitong Deng, Chaohui Zhao, Yanyan Li, Ting Sun, Youxin Zhou

**Affiliations:** ^1^ Neurosurgery & Brain and Nerve Research Laboratory The First Affiliated Hospital of Soochow University Suzhou Jiangsu P. R. China

**Keywords:** BET protein, BRD4, glioma‐initiating cells, notch1, self‐renewal, tumorigenicity

## Abstract

Bromodomain and extraterminal domain (BET) family proteins are considered to be epigenetic readers that regulate gene expression by recognizing acetyl lysine residues on histones and nonhistone chromatin factors and have been classified as curative targets for a variety of cancers. Glioma‐initiating cells (GICs), which commit self‐renewal, perpetual proliferation, multidirectional differentiation, and vigorous tumorigenicity, sustain the peculiar genetic and epigenetic diversification in the GBM patients, thus, GICs result in tumor recurrence. Abundant evidence demonstrates that BET proteins regulate differentiation of stem cells. However, it endures ambiguous how individual BET proteins take part in GIC advancement, and how do small molecule inhibitors like I‐BET151 target functional autonomous BET proteins. Here, we validated that BRD4, not BRD2 or BRD3, has value in targeted glioma therapy. We announce a signaling pathway concerning BRD4 and Notch1 that sustains the self‐renewal of GICs. Moreover, in‐depth mechanistic research showed that BRD4 was concentrated at the promoter region of Notch1 and may be involved in the process of tumor metabolism. Furthermore, in intracranial models, I‐BET151 eliminated U87 GICs’ tumorigenicity. The outcomes of this research could be conducive to design clinical trials for treatment of glioma based on BRD4.

## Graphical abstract

1. We evaluated BRD4, not BRD2 or BRD3, has value in targeted glioma therapy.

2. A signaling pathway involving BRD4 and Notch1 sustains GICs’ self‐renewal.

3. BRD4 was enriched at Notch1 promoter region and may be involved in the process of tumor metabolism.

4. BRD4 abolished the tumorigenicity of GICs in intracranial model after I‐BET151 (BET inhibitor) treatment.

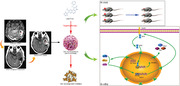

**Abbreviations**
BET: bromodomain and extraterminal domain
ESCs: embryonic stem cells
GBMs: glioblastomas
GICs: glioma‐initiating cells
GO: gene ontology
GSCs: glioma stem cells
Hes1: hes family bHLH transcription factor 1
KEGG: Kyoto Encyclopedia of Genes and Genomes
MACS: Model‐based Analysis of ChIP‐Seq
NICD: Notch intracellular domain
OS: overall survival
SOX2: SRY‐box transcription factor 2
STR: short tandem repeat

## 1 INTRODUCTION

Glioblastoma (GBM) is the most familiar and fatal primary tumor of the central nervous system. Based on maximal surgical resection, even as targeted therapy and immunotherapy have made progress, GBMs still have a poor prognosis.^1^ Due to the complexity caused by intratumoral molecular heterogeneity, targeted or customized therapies targeting specific subtypes or mutations mostly failed.^2^ Most tumors become resistant to treatment and quickly recur. Diverse studies have manifested the existence of cancer stem cells in many types of cancer consist of GBM.^3^, ^4^, ^5^ Glioma stem cells (GSCs), that is, glioma‐initiating cells (GICs), which commit self‐renewal, perpetual proliferation, multidirectional differentiation, and vigorous tumorigenicity, sustain the peculiar genetic and epigenetic diversification in the GBM patients.^4^, ^6^ Importantly, the resistance to chemotherapy and radiotherapy of GICs, which ultimately leads to tumor recurrence, recommends that GICs can be definitely targeted during therapy to diminish the jeopardy of tumor recurrence.^7^

Notch signaling is crucial for the regulation of GIC self‐renewal and tumorigenicity.^8^ Four Notch receptors, such as Notch1‐4 and five Notch ligands, including Jagged‐1/2 and Delta‐like‐1/3/4, have been recognized in mammals.^9^ When the Notch ligand binds to the notch receptor on adjacent cells, the Notch pathway is activated; this brings about the entry of Notch extracellular domain into signal‐sending cells through endocytosis.^10^ The Notch intracellular domain (NICD) is then discharged, translocated into the nucleus, and finally leading to Hey and Hes family genes’ upregulation.^11^ Even if initial treatment directing the Notch pathway can inhibit hypoxic tumor microenvironment formation and accelerate cell apoptosis, it is not beneficial to patients who received long‐term treatment for GBM.^12^, ^13^, ^14^, ^15^ Recent research on Notch1 in GICs has emerged. Mónica et al found that lipoprotein‐based nanostructures realize efficient GIC accumulation and therapeutic effects through CXCR4 receptor‐stimulated macropinocytosis, providing a powerful nanoplatform for RNA interference drugs targeting Notch1 to combat glioma.^16^ Research by the Bian XW team showed that positive feedback loop of NOTCH1‐SOX2 (SRY‐box transcription factor 2) manages the invasion of GSC along white matter bundles.^17^ Interestingly, the novel mechanism underlying Notch pathway‐dependent therapy is also discussed in this study.

Bromodomain and extraterminal domain (BET) family proteins are epigenetic readers that administer gene expression via recognizing acetylated lysine residues on histone and nonhistone chromatin factors.^18^, ^19^, ^20^ BRD4 is the most researched member of this family and is associated with multiple human cancers.^21^, ^22^, ^23^ Important research shows that cancer associated genes seem to be selectively dependent on BET proteins being targeting c‐MYC.^24^, ^25^ We attempted to discover the BET proteins in stem cells, especially in cancers. Recent research shows that H2A.Z.1 monoubiquitylation counters BRD2 to sustain chromatin balance in embryonic stem cells (ESCs).^26^ In addition, BRD4 dominates the development of hematopoietic stem cells and regulates the inflammatory response of macrophages.^27^ In cancer stem cells, several studies mentioned that targeting BRD2 and BRD4 inhibited GIC proliferation and BRD4‐depleted induced GIC apoptosis.^28^, ^29^ However, it is not so clear that the molecular mechanism by which BET protein take part in GIC processes. Interestingly, a recent study reported that BRD4 modulates the spread of breast cancer by Notch1/Jagged1 signaling.^30^ We develop a hypothesis based on these insights that BET family proteins, especially BRD4, can be effective to the regulation of self‐renewal and tumorigenicity of GIC.

In this study, we assessed the relationship between the BRD4 and Notch1 pathways in the situation of GIC self‐renewal. Our study suggested firstly that confronting BRD4 inhibits self‐renewal and tumorigenicity of GIC by modulating Notch1 signaling. Further research has shown that BRD4 associates with a region of the Notch1 promoter. Therefore, targeting BRD4 could be an encouraging medical strategy to prevent the progression of GBM.

## 2 MATERIALS AND METHODS

### 2.1 Cell culture

Glioma cells, including U87 and U251, were attained from the Shanghai Institutes for Biological Sciences. Primary cells were obtained from tumor tissue of GBM patients in the First Affiliated Hospital of Soochow University.^31^ Primary cell line used in this study has been identified by Short Tandem Repeat (STR), but relevant test results are not yet published. Cells or GICs were cultured in DMEM medium as previously described. The methods of isolation and identification of GBM initiating cells were found in our previous study. The model of CD133+ glioma was established. First, magnetically activated cell sorting (MACS) was used to collect CD133+ cells from glioma cells. CD133+ cells in MACS+ population were then quantitatively analyzed using flow cytometry to test the validity of sorting. Stem cell culture medium was used to culture CD133+ cells to form nerve spheres, while GBM cells not classified as CD133 positive could not form spheroids under the equivalent culture conditions.

### 2.2 CCK8, neurosphere formation, and limiting dilution assay

The cell suspension was evenly spread on a 96‐well plate with a volume of about 50 µL. According to different experimental requirements, another 50 µL containing drugs were added, with a total volume of 100 µL. Or 100 µL of stable lentivirus transfected cell suspension was added directly to the 96‐well plate. For CCK8, 10 µL of CCK8 (Dojindo, Japan) reagent was added. After 2 h in a constant temperature incubator at 37°C, the optical density value was measured by an enzyme marker at the wavelength of 450 nm, and the cell viability was evaluated by the difference of the optical density value. This method can also be used to detect cell proliferation. In the Neurosphere formation experiment, cell counts and images were taken under an inverted microscope for easy quantification. For the limiting dilution assay, medium with different drug concentration was configured first. After counting the total stem cell suspension, different cells were added to the prepared medium for blending, and then added to the 96‐well plate to reduce the error in the repeated experiment. Finally, the cell ball was counted. Detailed experimental methods can be seen in previous studies.^32^

### 2.3 Drug treatments and lentiviral shRNA transfection

For I‐BET151 (Selleckchem #S2780, US) treatment and DAPT(GSI‐IX) (Selleckchem #S2215, US) treatment, in tumor sphere formation, Western blot, limiting dilution experiment and cell viability assay, GBM initiating cells were cultured with I‐BET151 (0, 2, 4 µM) and (0, 20, 40 µM) for 24 h, respectively or DAPT (10 µM) for 24 h. U87 and U251, primary initiating cells were cultured with I‐BET151(0, 4 µM) and (0, 40 µM) for 24 h, respectively or DAPT (10 µM) for 24 h in Immunofluorescence analysis. Lentiviral shRNAs against the BRD2/BRD3/BRD4/Notch1 genes were produced by GV112 vector (hU6‐MCS‐CMV‐Puromycin; GeneChem, China). BRD4 overexpression lentivirus was prepared using GV692 vector (Ubi‐MCS‐3FLAG‐CBh‐gcGFP‐IRES‐puromycin; GeneChem). On the basis of the manufacturer's recommendation, lentiviral vectors expressing shRNA or scrambled transfected into cells. Steady cell clones transfected with shRNA expressing constructs were chosen with puromycin intervention after infection.

### 2.4 Immunohistochemistry

The antibiotin protein‐biotin method got accustomed to perform immunostaining on paraffin‐embedded sections. Slides were dewaxed in xylene, then rehydrated in graded ethanol, then the endogenous peroxidase activity was then quenched with 0.3% hydrogen peroxide (China), and the strong antigen recovery solution was heated to 37°C to recover the antigen. A total of 5% goat serum (Solarbio, China) was used to block nonspecific proteins. Primary antibodies (1:100 dilutions) were used to incubate sections at 4°C overnight, subsequently the appropriate biotinylated secondary antibody was added (1:100 dilutions) (ZSGBBio, China) at 37°Cfor 60 min. Then, slides were then hatched with ABCperoxidase and diaminobenzidine (ZSGBBio). Next, the slides were counter‐stained for nuclear staining by Mayer hematoxylin solution (Solarbio). For going on H&E staining, the slides were deparaffinized and rehydrated. Next, the slides were stained by nuclear staining, subsequently restaining using HE kit (Solarbio). The images were taken with an inverted microscope (Olympus, Japan). The human tissue samples used in this study research have complied with the relevant national and institutional policies.

### 2.5 Western blot and immunofluorescence

For Western blot, after the cell protein sample was extracted, the protein concentration was detected by the BCA kit (Beyotime, China) and balanced, and a one of three proportion of loading bufffer was added for high temperature (100°C) denaturing for 15 min. Then, the samples were added to the glue‐plate sample adding tank. Electrophoresis was carried out at constant voltage for about 90 min, and then membrane was carried out at constant current for about 90 min. Then, it was sealed with milk, incubated with primary antibody at 4°C overnight, and then exposed after incubation with secondary antibody the next day. For immunofluorescence, cell spheres or stem cell suspensions were fixed with 4% paraformaldehyde in a 15 mL centrifuge tube, air‐dried slides were placed on adhesive slides, 5% BSA was sealed, primary antibody was dropped and placed in a wet box at 4°C overnight. Fluorescent secondary antibodies were incubated the next day and sealed with DAPI sealing tablets. Images were then taken with a confocal microscope. Specific experimental methods have also been used in previous studies.^32^

### 2.6 qRT‐PCR

Trizol assay was used to obtain total RNA. Reverse transcription reactions were performed on 1 µg of RNA with the NovoScript® Plus All‐in‐one 1st Strand cDNA Synthesis SuperMix (gDNA Purge) kit (E047, Novoprotein, China). The primers sequences are listed in Supporting information Table S3. PCR amplifications were performed with a direct RNA qPCR MasterMix (E198, Novoprotein) on the ABI Prism 7500 heat cycle apparatus.

### 2.7 Chromatin immunoprecipitation

U251 GICs were treated with I‐BET151 and shBRD4. According to the instructions of the Chromatin IP kit (Cell Signaling # 56383S), cells were fasten in 1% formaldehyde at 37°C for 15 min and terminated with glycine (125 mM) for 10 min, then completed cell cross‐linking, then prepared nuclei, and sonic fragmented chromatin by Bioruptor (Diagenode). Chromatin was precipitated with anti‐mouse IgG or anti‐BRD4 (10 µL) with Dynabead magnetic beads. Each sample was evaluated by qPCR in triplicate. Each a Chromatin Immunoprecipitation (CHIP) DNA fragment's *Ct* value was standardized to the input DNA fragment's *Ct* value detected by the same qPCR Assay (Δ*Ct*) to illustrate the difference in chromatin sample preparation. Calculate the %Input for each ChIP fraction as 2^ [*Ct* (input) – *Ct* (ChIP)] × *Fd* × 100% (*Fd* is input dilution factor, equal to 100/5 = 20), and anti‐IgG fold enrichment was evaluated. ChIP primers sequences used are exhibited in Supporting information Table S3.

### 2.8 CHIP‐seq and analysis

As mentioned above, the cells were lysed, sonicated, and used in ChIP reaction as per the manufacturer's recommendation. The ChIP processed DNA was then reverse cross‐linked and purified. The ChIP DNA was quantified and used for construction of next generation sequencing libraries. Analysis of Sequencing Data includes the capital stages of DNA sequencing, including image processing, base calling, quality control, and alignment. BOWTIE software (Version 2.1.0) was used to align the readings with the human genome (HG19). For peak detection, the mapped readings were applied to MACS (Model‐based Analysis of ChIP‐Seq) Version 1.4.2 software peak detection. Statistically obvious ChIP concentration regions (peaks) were assessed by comparing IP with Input or with Poisson background model (Cut‐off *P* value = .05). Peaks detected by MACS contains peaks in samples. Peak annotation contains the nearest gene annotation of the peaks using the newest UCSC RefSeq database. Promoter‐centered annotation is the nearest gene annotation containing the peaks in the range of −2 Kb to +2 Kb around the corresponding gene TSS using UCSC RefSeq database. GO analysis is a GO term analysis of genes whose promoter region (−2 Kb to +2 Kb around the TSS) contains the peaks. Pathway analysis is a KEGG pathway analysis of genes whose promoter region (−2 Kb to +2 Kb around the TSS) contain the peaks. Data are visualized by the chip sequence diagram of each sample in the UCSC Genome Browser.

### 2.9 Nude mouse tumor model

U87 cells were used to establish a model of intracranial tumor in Female nude mice, refer to previous studies for details.^32^ The cells that had been transfected with luciferase encoding lentivirus (GeneChem) were stereotaxically injected into the intracranial of mice (n = 6 in each group) to establish tumor models. The mice were randomly divided into two groups. The drug is soluble in 20% dimethyl sulfoxide, 40% PEG‐400, and 40% PBS. Mice that survived were intraperitoneally injected with I‐BET151 (16 mg/kg/day) and DMSO 3 days a week 7 days after cell inoculation. At day 7, 14, and 28, intracranial tumor size was assessed using the IVIS spectral real‐time imaging system (Blandford, USA). Animal research is conducted in accordance with internationally recognized norms and national regulations. For HE and IHC, mice brains were immobilized in 4% paraformaldehyde and then embedded in paraffin.

### 2.10 Statistical analysis

The bar chart is represented by mean standard deviation from at least three experimental replicates. Most of the experiments were statistically analyzed using Student's *t* test. One‐way analysis of variance (ANOVA) followed by Tukey's post‐hoc test was used to assess differences between groups. The data were analyzed by graphpad prism 6. Significance of *P* values were set at ^NS^ *P* > .05, *P < .05, **P < .01, ***P < .001, ****P < .0001.

## 3 RESULTS

### 3.1 BRD4 mRNA is negatively associated with overall survival (OS) in glioma patients

A total of 680 TCGA RNA‐s Equation (TCGA‐seq) samples and 281 CGGA RNA‐s Equation (CGGA‐seq) samples were evaluated to investigate the relation between mRNA expression of BET family members BRD2/BRD3/BRD4, and overall survival (OS) in glioma patients. In TCGA, OS differences between the BRD2/BRD3/BRD4 groups with high and low mRNA expression were constantly significant (*P* < .0001). We found that higher expression of BRD2/BRD3 and lower expression of BRD4 was associated with consistently higher OS (Figure 1A). In CGGA, lower expression of BRD2/BRD4 and higher expression of BRD3 in the primary glioma datasets was associated with survival superiority of patients (Figure 1B). In recurrent glioma, BRD4 expression (*P* < .05), but not BRD2/BRD3 expression (*P* > .05), resulted in significant differences in OS (Figure 1C).

FIGURE 1BRD4 mRNA is inversely correlated with OS in patients with glioma and relates to glioma histology and GBM subtype. (A‐B) BRD2, BRD3, BRD4 correlated with OS of patients with glioma. TCGAs Equation (A), CGGAs Equation (B, C) datasets were used for survival analysis in primary/recurrent glioma/glioma. The median of the data was sampled to distinguish BRD low‐ and high‐expression in patient samples. (D‐E) CGGAseq and GSE16011mic datasets were used to estimate the correlation between BRD4 mRNA expression and tumor grade (WHO grade). (E) mRNAs of different histologies were examined with CGGAseq and GSE16011mic datasets, which oligodendroglioma (O), anaplastic oligodendroglioma (AO), oligoastrocytoma (OA), anaplastic oligoastrocytoma (AOA), astrocytoma (A), anaplastic astrocytoma (AA), and GBM. (F) The expression features in GBM subtypes were also explored with the TCGA and CGGA datasets. (G ,H) Protein expressions of BRD4 in normal and tumor tissues were detected by western blot. Data are shown as means ± SD, *n* = 5, #P = NS, *P < .05,**P < .01, ***P < .001, ****P < .0001, Student's *t*‐test. (I) IHC staining in normal and tumor tissues (× 40 magnification, scale bar = 200 µm)

**Figure:**
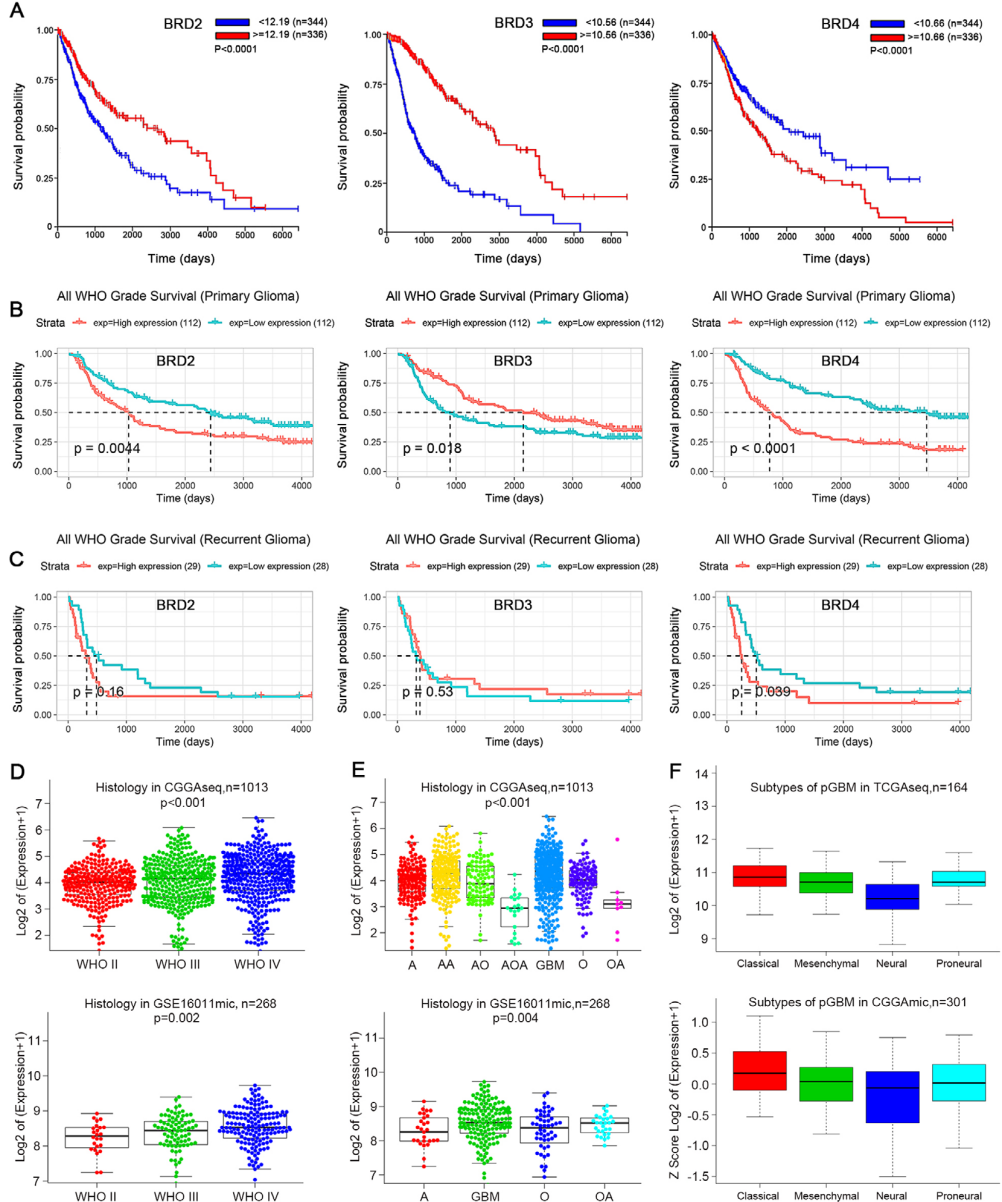

**Figure:**
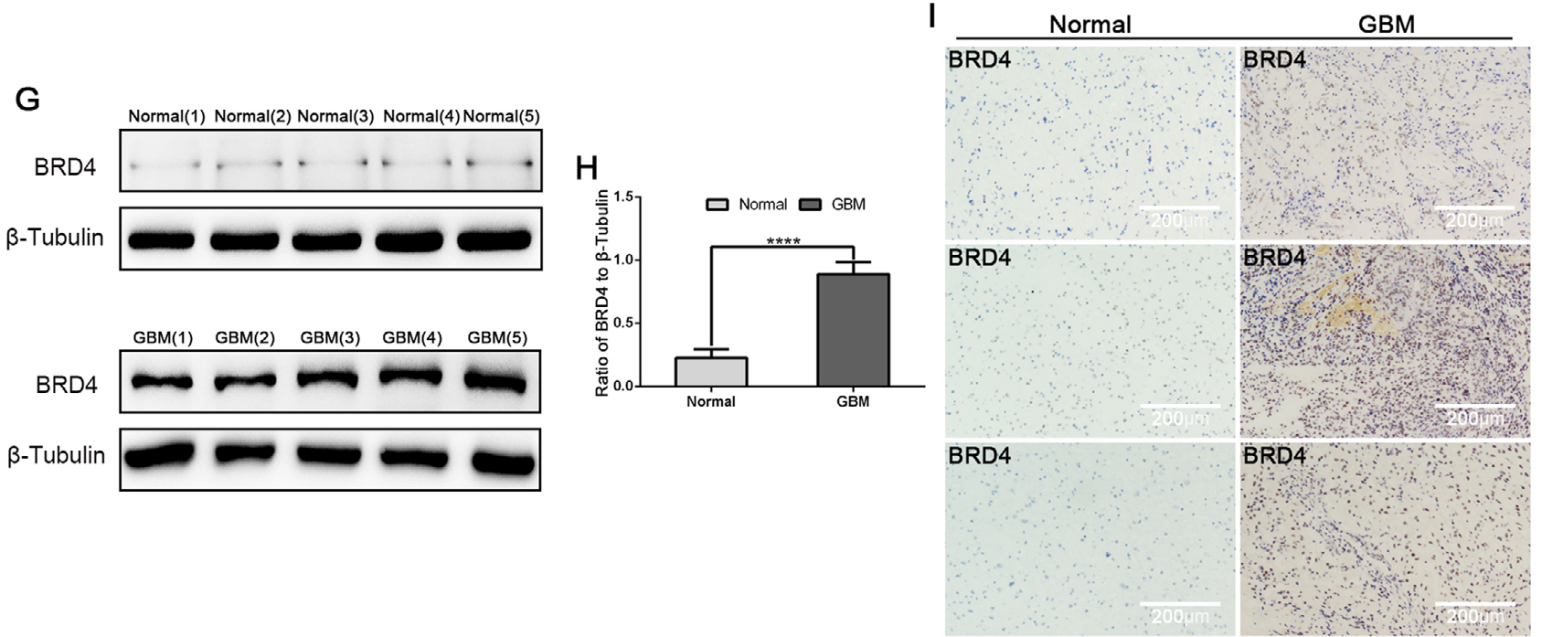

### 3.2 The mRNA expression of BRD4 associates with glioma histology and GBM subtype

We then investigated BRD4 expression in glioma in 1013 CGGA samples and 268 GSE16011 samples. We delved in the association between BRD4 expression and histology of glioma in the genomic datasets (Figure 1D–F). Histological subtypes included astrocytoma (A), anaplastic oligodendroglioma (AO), oligodendroglioma (O), anaplastic oligoastrocytoma (AOA), oligoastrocytoma (OA), anaplastic astrocytoma (AA), and GBM. The mRNA expression of BRD4 was identified to be associated with tumor grade (WHO grade) (Figure 1D); BRD4 has the highest expression in GBM (Figure 1E). We found that the mRNA expression of BRD4 was higher in the proneural and classical subtypes of glioma than in the mesenchymal and neural subtypes (Figure 1F). In addition, we detected BRD4 expression in normal and tumor tissues, and the results made clear that BRD4 expression in tumor tissues is undoubtedly higher than that in normal tissues (Figure 1G–I). These results suggest BRD4 is implicated in GBM.

### 3.3 BET protein inhibition reduces self‐renewal and proliferation of GIC in vitro

In view of exposed and preliminary data, we conjectured that BET proteins modulate the GIC stemness phenotype.^33^, ^34^ I‐BET151, a novel selective BET inhibitor for BRD2, BRD3, and BRD4, showed extensive research in cancer,^35^, ^36^ was selected^37^; thus far, little research has been done on the role of I‐BET151 in glioma. I‐BET151 does not inhibit BRD2, BRD3, and BRD4 expression, but it affects how they bind to acetylated histone peptides (Supporting information Figure S1C and D).^37^

The effects of I‐BET151 on GIC survival were appraised using Cell Counting Kit‐8. I‐BET151 treated with different concentrations for different periods reduced cell viability (Supporting information Figure S1A and B). Previous researches revealed that I‐BET151 is effective for 24 h in U87 and U251 cells.^38^ The IC50 value of I‐BET151 treatment for 24 h is about 10 µM in U87, 100 µM in U251, and 95 µM in primary cells. According to IC50 values, I‐BET151 at concentrations of 0, 2, and 4 µM was used in subsequent experiments (when U87 cell was treated for 24 h) and 0, 20, and 40 M (when U251 or primary cell was treated for 24 h).

**FIGURE 2:**
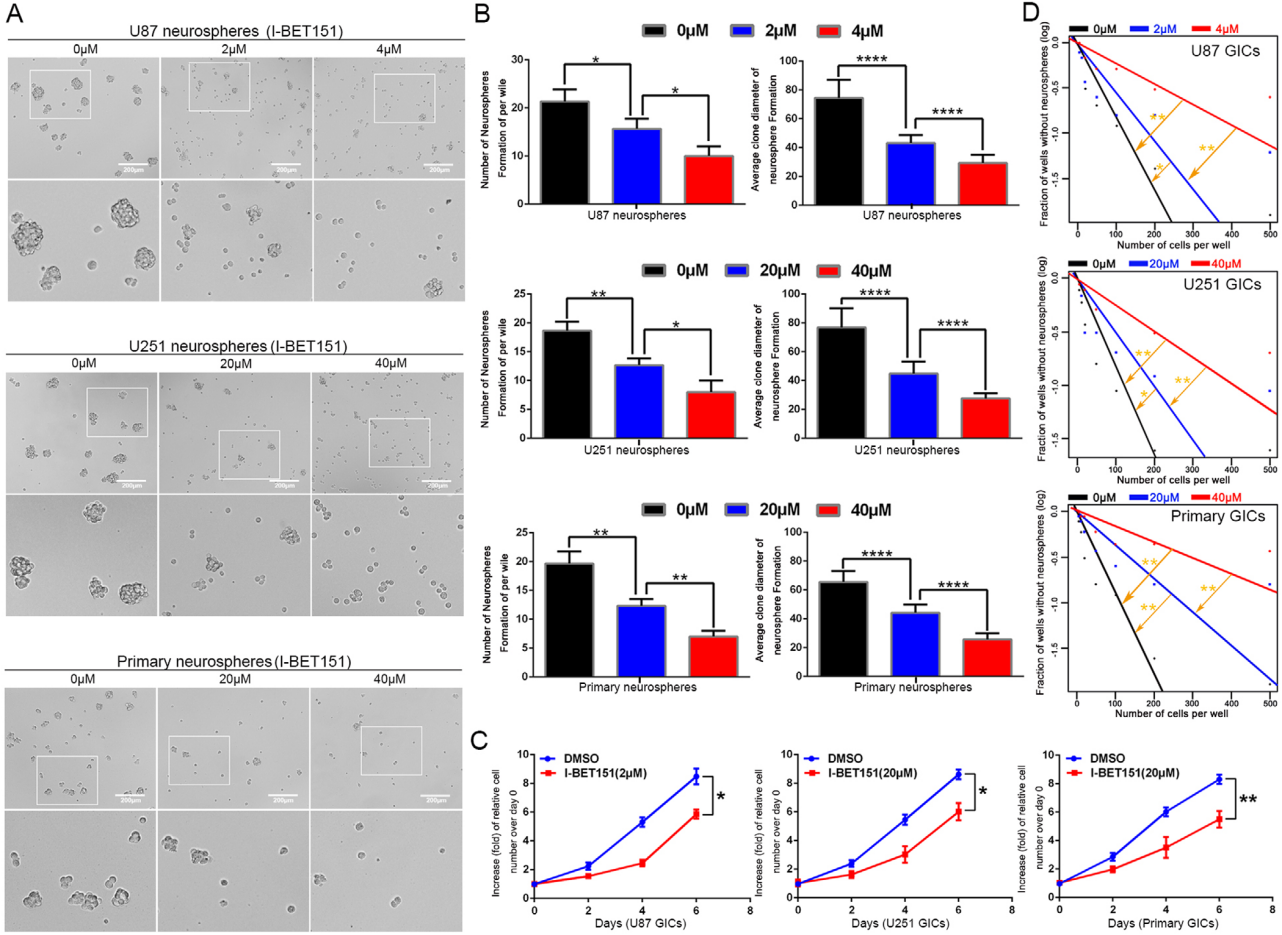
Effects of GIC self‐renewal and proliferation treated with I‐BET151 in vitro. (A) The representative images of GICs neurospheres showed that neurosphere formation ability of GICs was significantly inhibited by I‐BET151 treatment. The outlined sections of top images were defined as higher magnification sections below. *n* = 5, scale bar = 200 µm. (B) The quantification of numbers and diameter of the GICs neurospheres showing that neurosphere formation ability of GICs were obviously inhibited after I‐BET151 treatment. (C) The ability of GICs proliferation was showed by cell viability assay. (D) The ability of GICs self‐renewal was detected by in vitro limiting dilution assay. Data are shown as means ± SD, n = 5, *P < .05, **P < .01, likelihood ratio test. Data in B, C are shown as means ± SD, n = 5, *P < .05, **P < .01, ****P < .0001, Student's *t*‐test

Limiting dilution, neurosphere formation, and cell viability assays were used to clarify whether BET proteins regulate GIC self‐renewal and proliferation.^39^ The neurosphere formation assay showed that I‐BET151 effectually intercepted GIC neurosphere formation (Figure 2A). We demonstrated the effectiveness of treatment with I‐BET151 by measuring and quantifying the number and size of the tumor spheres from U87, U251, and primary cells (Figure 2B). Cell viability assay determined that I‐BET151 reduced proliferation of GIC (Figure 2C). For limiting dilution assays, the proportion of foramen lacking tumor spheres increased continuously with the increase of I‐BET151 concentration. Compared to the control group, I‐BET151 treatment certainly increasedU87, U251, and primary GICs’ self‐renewal (Figure 2D).

### 3.4 Targeting BRD4 inhibits self‐renewal of GIC in vitro

To further study the BET proteins, we used a special shRNA knockout strategy to probe the requirements for BRD2, BRD3, and BRD4. Previous experiments showed that GBM cells express BRD2, BRD3, and BRD4 in this study (Supporting information Figure S1C). We evaluated each BET members’ mRNA expression to verify the knockdown efficiency and specificity of each BET family members’ mRNA expression. We certified that each shRNA only destroyed its target sequence without decreasing the mRNAs of other BET family members, especially BRD4, more effective knocking down of shBRD4 (1) or shBRD4 (3) were used in following studies (Supporting information Figure S1G). Transfection with shRNA for 72 h resulted in selective consumption of each protein (Supporting information Figure S1E and F). Then, we examined self‐renewal and proliferation of BET protein depleted GICs in the neurosphere formation limiting dilution, and CCK8 assays. Neither BRD2 nor BRD3 elimination modulated self‐renewal. Significantly, BRD4 elimination decreased self‐renewal (Supporting information Figure S2A‐B, D). Interestingly, BRD2 or BRD4 depletion led to reduced cell proliferation, but BRD3 did not (Supporting information Figure S2C). CD133, Nestin, and SOX2, the expressions of these stem cell markers, were also detected in U87, U251, and primary GICs. I‐BET151 treatments and BRD4 depletion decreased Nestin, CD133, and SOX2 at the protein level (Supporting information Figure S3A‐C). Neither BRD2 nor BRD3 interfered with the expression of CD133, Nestin, and SOX2 in GBM cells (Supporting information Figure S4A‐C). As shown by western blotting and immunofluorescence staining, these treatments suppressed GIC stemness maintenance. Taken together, our research newly confirmed BRD4 was involved in the regulation of GIC self‐renewal and proliferation.

### 3.5 BRD4 regulates GIC self‐renewal and proliferation through Notch1 signaling

Based on the above observation, it revealed that GIC self‐renewal was regulated by BRD4. To explore the underlying mechanism, we paid attention to the Notch1 pathway, which is supposed to be a pivotal manager of maintaining the stemness phenotype of GIC.^17^ Activation of the Notch pathway has been shown to lead to NICD translocated into nucleus and activate Hes1 (hes family bHLH transcription factor 1) transcription.^40^ Our study indicated that Notch1 and Hes1 expression of GICs was attenuated after BRD4 inhibition by immunofluorescence staining (Figure 3A). We showed that, when inhibited by I‐BET151, BRD4 significantly downregulates Notch1/NICD/Hes1 expression in a dose‐dependent manner, and the BRD4 depletion groups showed the same result (Figure 3B and C), neither BRD2 nor BRD3 interfered with Notch1, NICD, and Hes1 expressions in GBM cells (Supporting information Figure S4A‐C). These results suggest that inhibiting BRD4 decreased Notch1 pathway activation.

FIGURE 3BRD4 regulated the protein expression of Notch pathway. Cells were treated with I‐BET151 and shBRD4 knockdown.(A) Immunofluorescence staining of GICs, which treated by DMSO, I‐BET151(4/40 µM) and scramble, shBRD4(1). (B,C) Protein expressions of Notch1 pathway were detected by western blot. Data are shown as mean ± SD, n = 3, #P = NS, *P < .05,**P < .01, ***P < .001, ****P < .0001, Student's *t*‐test. The nuclei were stained with DAPI and the antibody against Notch1 and Hes1. Images were captured by laser confocal microscope (× 400), scale bar = 20 µm

**Figure:**
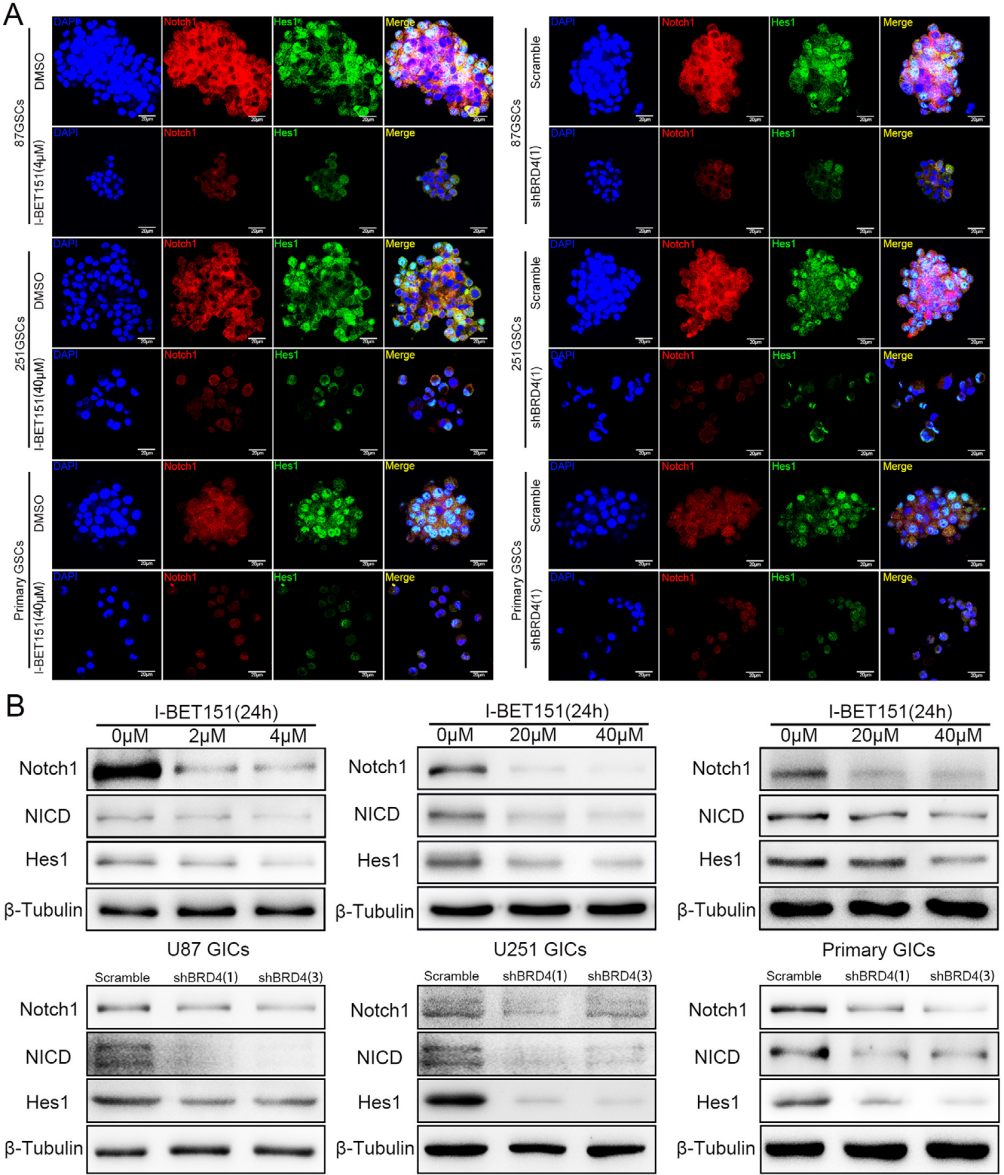

**Figure:**
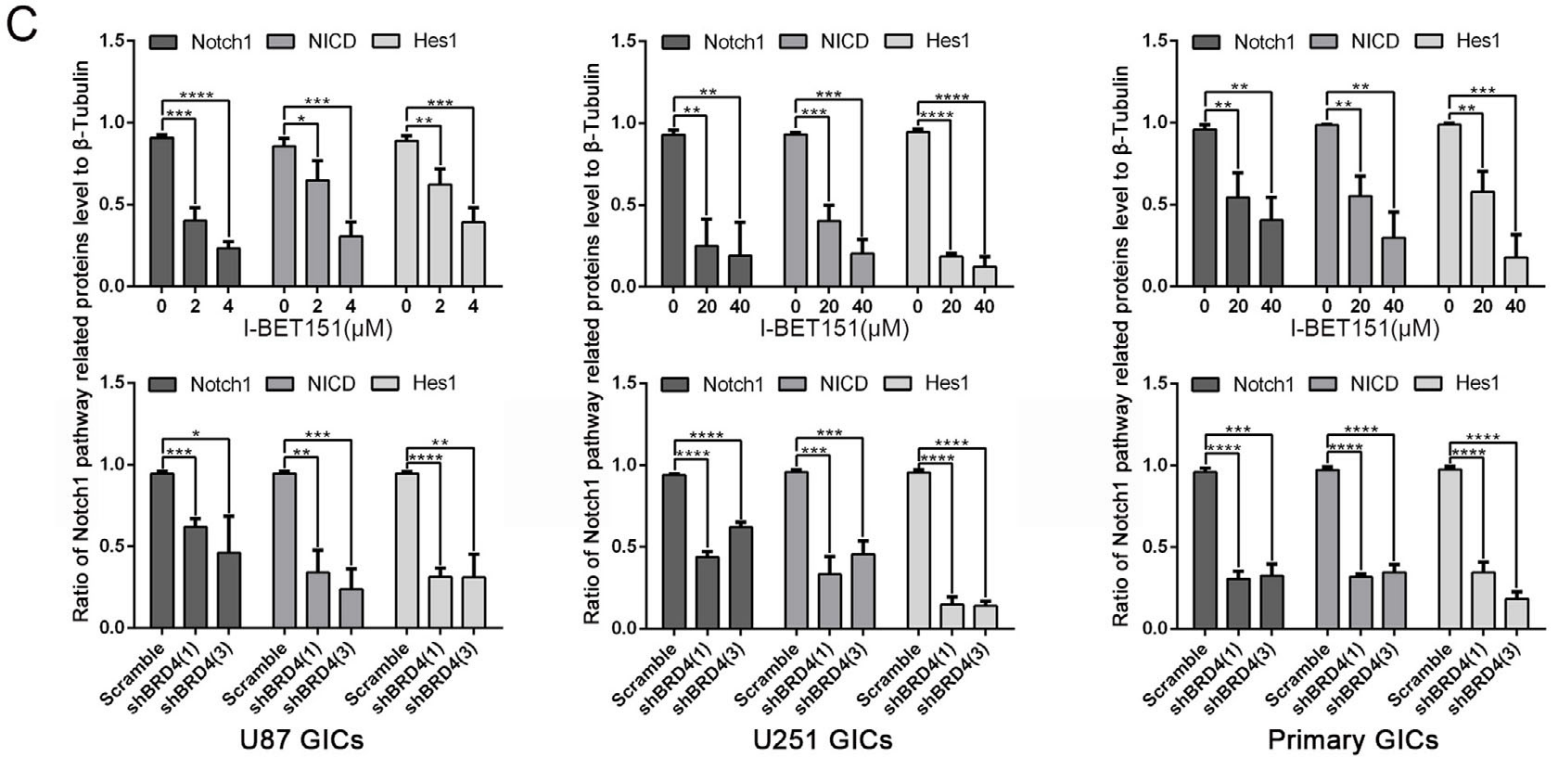

**FIGURE 4:**
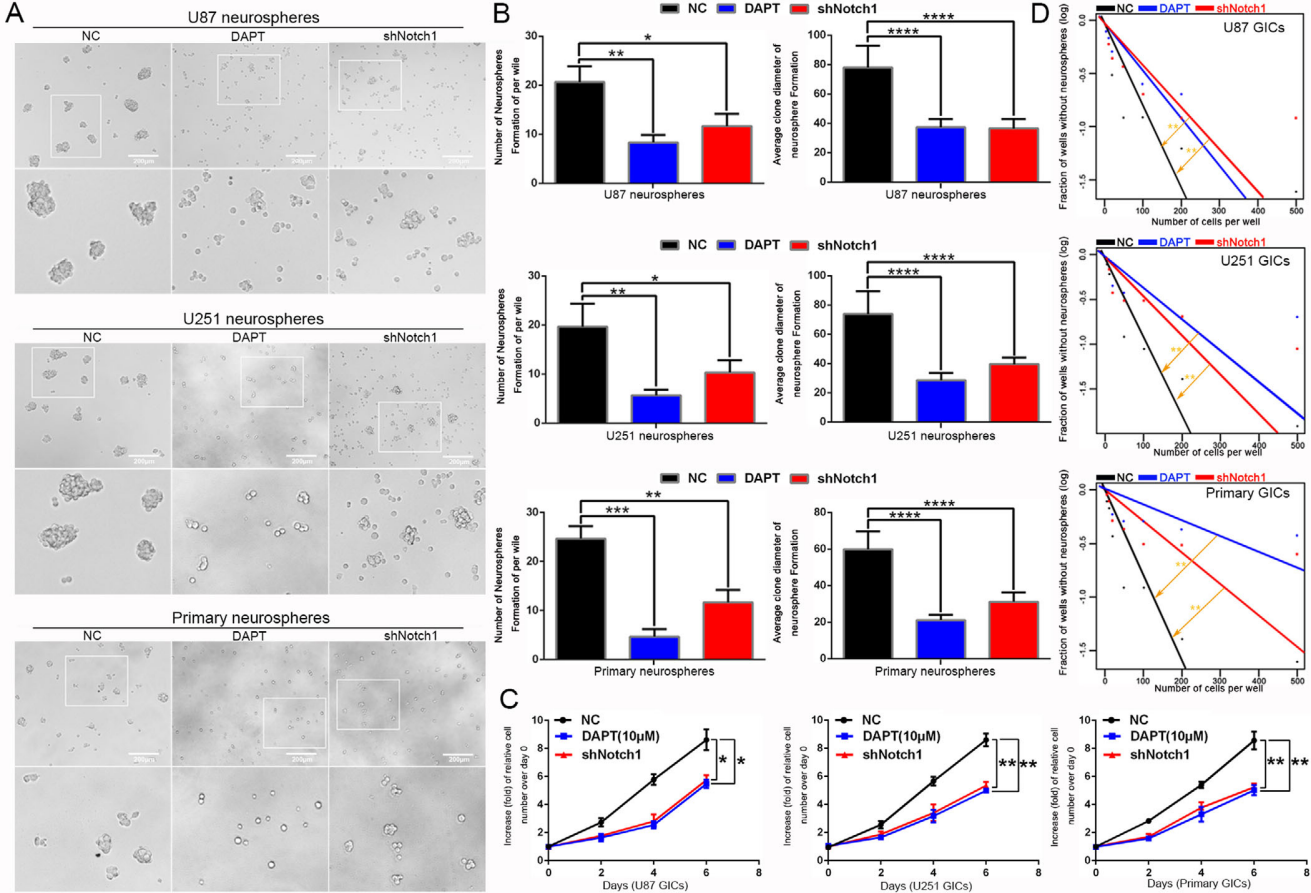
Impacts of GIC self‐renewal and proliferation treated by Notch1 inhibition in BRD4 overexpression GICs. (A) The representative images of GICs neurospheres showed that neurosphere formation ability of GICs was significantly inhibited by DAPT (10 µM) treatment and shNotch1 knockdown. The outlined sections of top images were defined as higher magnification sections below. n = 5, Scale bar = 200 µm. (B) The quantification of numbers and diameter of the GICs neurospheres showing that neurosphere formation ability of GICs were obviously inhibited after DAPT treatment and shNotch1 knockdown. (C) The ability of GICs proliferation was showed by cell viability assay. (D) The ability of GICs self‐renewal was detected by in vitro limiting dilution assay. Data are shown as means ± SD, n = 5, **P < .01, likelihood ratio test. Data in B, C are shown as means ± SD, n = 5, *P < .05, **P < .01, ***P < .001, ****P < .0001, Student's *t*‐test

**FIGURE 5:**
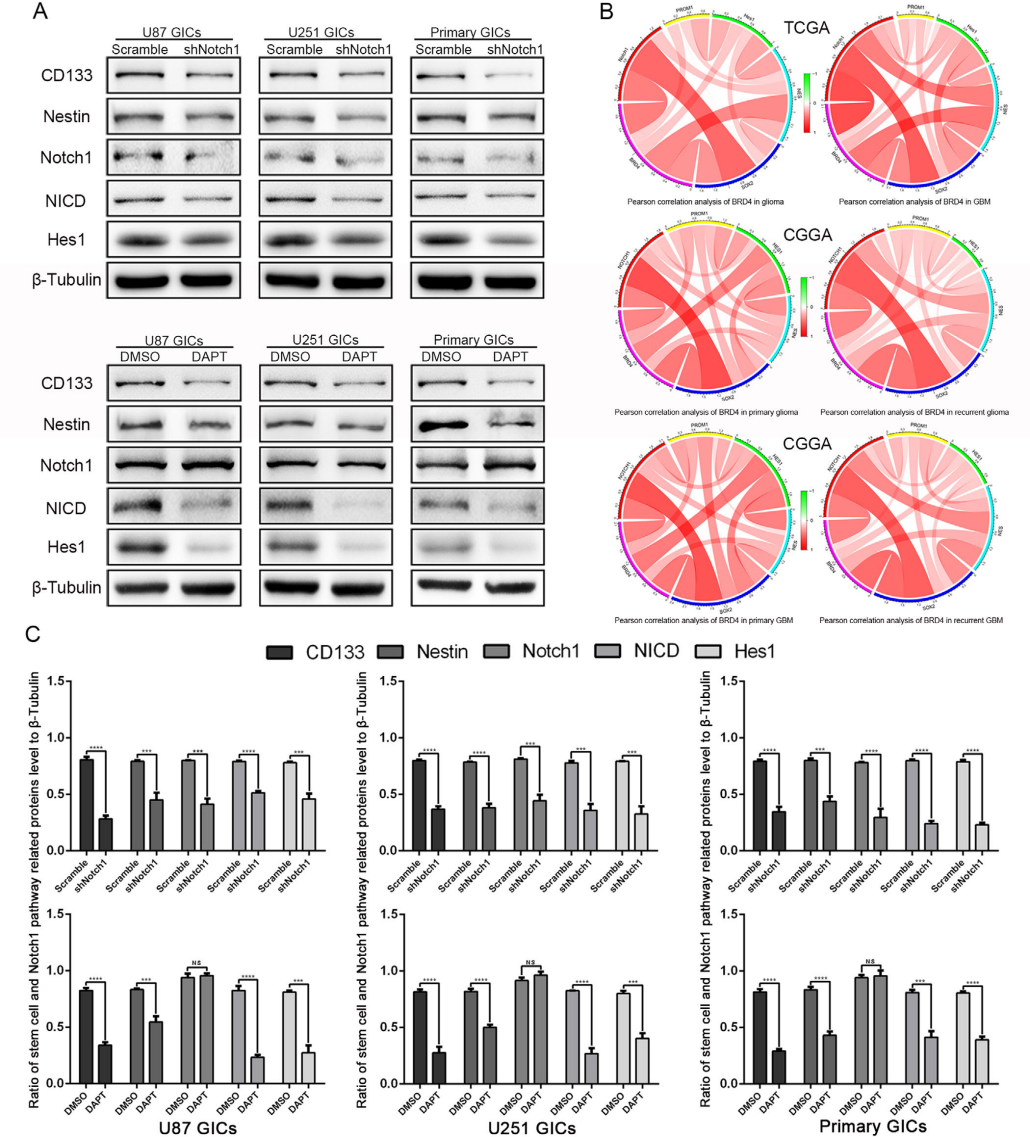
Notch1 regulated the protein expression of stem cell markers in BRD4 overexpression GICs, and Pearson correlation analysis of BRD4 with Notch1, Hes1, Nestin, CD133, and SOX2 in TCGA and CGGA databases. (A) Cells were treated the same as in (Picture 4). (A,C) Protein expressions of Notch1 pathway and stem cell markers were detect by western blot. Data are shown as means ± SD, n = 3, #P = NS, *P < .05,**P < .01, ***P < .001, ****P < .0001, Student's *t*‐test. (B) Pearson correlation analysis between BRD4 and Notch1 pathway, stem cell markers in TCGA and CGGA data sets

Since targeting BRD4 inhibited the self‐renewal of GICs and abolished Notch1 pathway activation, we speculated that BRD4 regulates the self‐renewal of GICs through Notch1 signaling. This is supported by the fact that using the novel y‐secretase inhibitor, DAPT (GSI‐IX), to inhibit Notch1 in BRD4 overexpressing cells still inhibits GIC self‐renewal and proliferation.^41^ We discovered that Notch1 suppressed by DAPT and shRNA still inhibited GIC self‐renewal and proliferation in BRD4 overexpressing cells (Figure 4A‐D). We also found that Nestin, CD133, NICD, and Hes1 expressions were reduced after DAPT therapy and Notch1 knocking down (Figure 5A and C). We found that CD133 and Nestin expression in GICs were abrogated after Notch1 pathway inhibition by Immunofluorescence staining (Supporting information Figure S5A). These results show that overexpression of BRD4 fails to stop the downregulation of the GIC stemness phenotype, which is caused by targeting Notch1 signaling. We also evaluated the Pearson correlation between BRD4 and Notch1, Hes1, Nestin, CD133, and SOX2 in the TCGA and CGGA databases. The data showed that BRD4 had a stronger correlation with Notch1, Hes1, Nestin, and SOX2. The low correlation with CD133 could be that the PROM1 gene did not express CD133 (Figure 5B). Refer to Supporting information Figures S5 and S6A and B for specific data.

### 3.6 BRD4 bonding to the Notch1 promoter region

**FIGURE 6:**
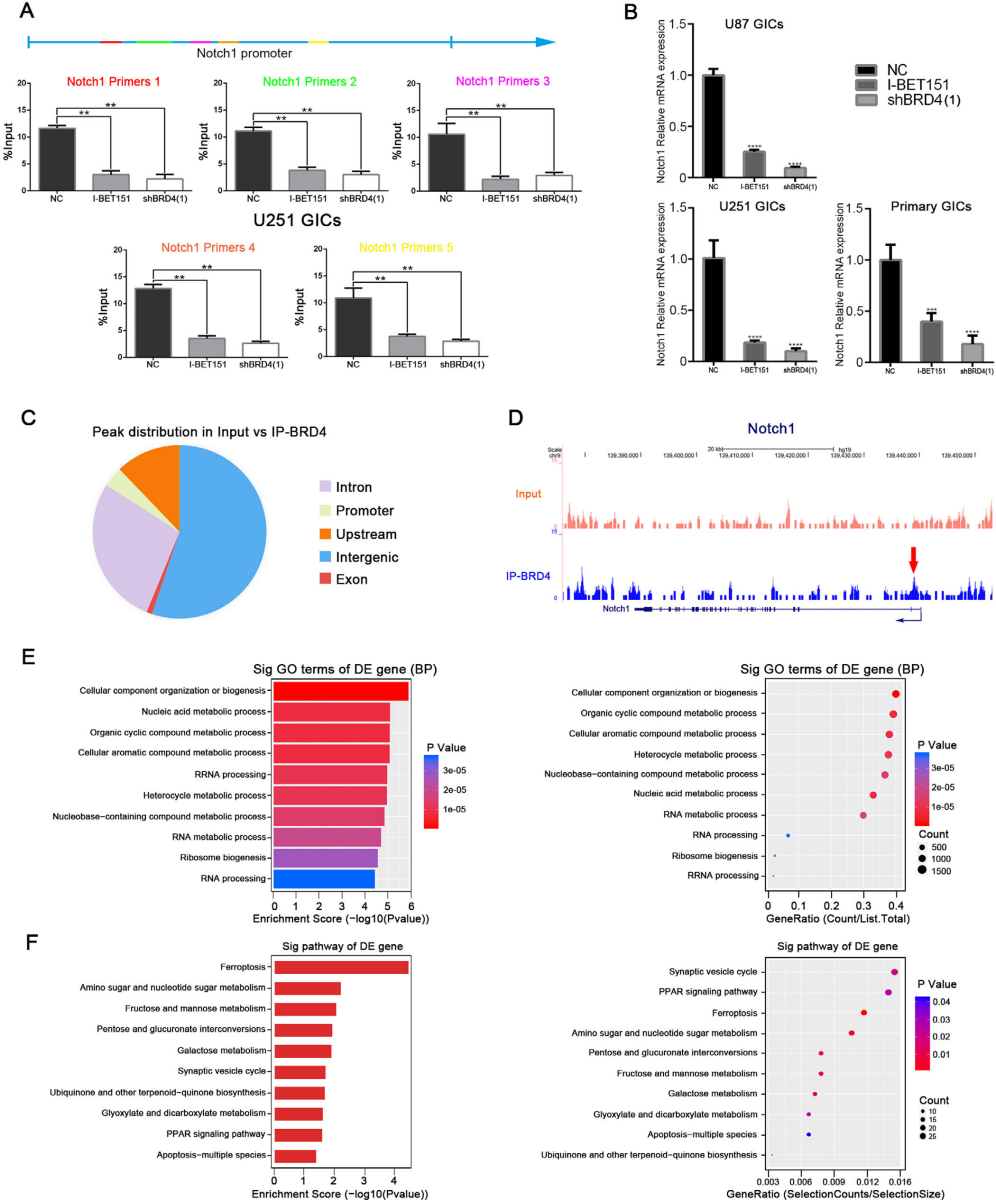
BRD4 was enriched in the Notch1 promoter region and occupied cell metabolism genes regulatory regions in U251 GICs. (A) BRD4 ChIP qPCR at promoters of Notch1. IgG was used as a reference, n = 3. (B) The effect of silencing Notch1 was validated by RT‐PCR. Statistical analysis was performed by using one‐way ANOVA followed by Tukey's post‐hoc test. Data represent means ± SEM. **P < .01. (C) Pie chart showing BRD4 distribution in input versus IP‐BRD4. (D) BRD4 ChIP‐seq tracks of Notch1gene. Bottom to top: IP‐BRD4, Input. (E‐F) Gene ontology analysis and KEGG pathway analysis of genes with higher BRD4 enrichment in U251 GICs of Input versus IP‐BRD4

**FIGURE 7:**
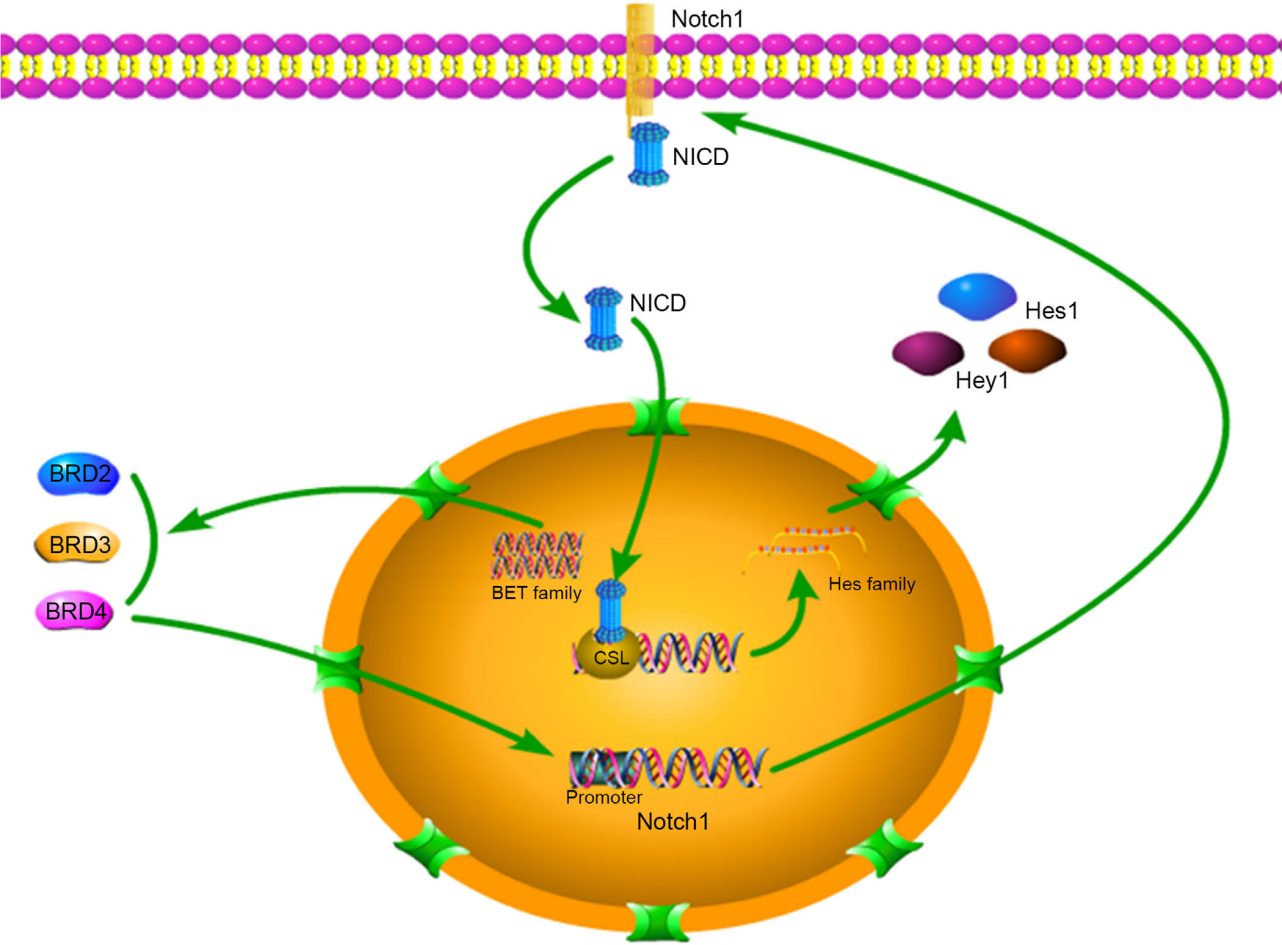
Mechanism diagram described the line of Notch1 pathway activation and the progress of BRD4 regulating Notch1 promoter

**FIGURE 8:**
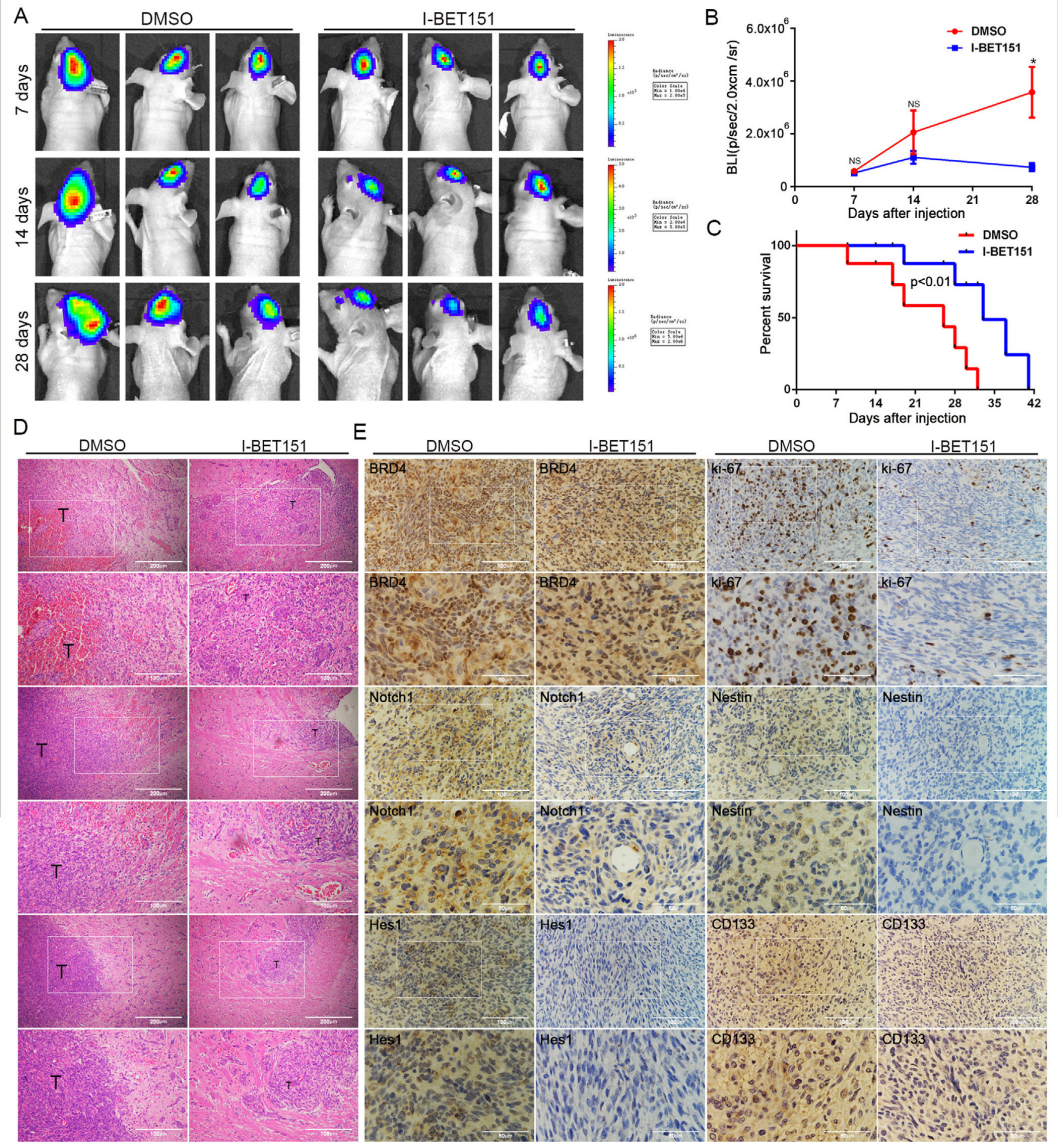
I‐BET151 inhibited the tumorigenicity of U87‐MG GICs in vivo. The mice were treated with intraperitoneal injection with DMSO, I‐BET151 (16 mg/kg/day) for 3 days a week. The treatment started from the 7th day after implantation and lasted for approximately 28 days. (A) Representative images of bioluminescence of mice on days 7, 14, and 28 after implantation. (B) Quantitative analysis of these bioluminescence images for the DMSO, I‐BET151 treatment groups. Data are shown as the mean ± SD, n = 6, **P < .01 compared to the control, Student's *t*‐test (C) The overall survival of mice in the DMSO, I‐BET151 treatment groups. Data are shown as the mean ± SD, n = 6, ^NS^ *P* > .05, **P < .01 compared to the control, ANOVA test. (D‐E) Representative images of the HE (× 40 magnification, scale bar = 200 µm) and IHC staining in tumor sections (× 100 magnification, scale bar = 100 µm).The three rows of HE samples are repeated data from different processing groups. The outlined sections of top images were defined as higher magnification sections below

As shown above, to precisely determine the molecular contact between BRD4 and Notch1, we explored Notch1 expression in both control cells and BRD4 eliminated or inhibited cells; BRD4 inhibition and depletion both led to Notch1 downregulation (Figure 8A and B). To elucidate the molecular mechanisms of this management, we used ChIP to assess whether BRD4 related to the Notch1 promoter. We designed five primer sequences for the Notch1 promoter region. I‐BET151 destroys BET protein/chromatin interactions by competing for acetylated histone binding sites. Interestingly, I‐BET151 treatment prevented the relation of BRD4 with the Notch1 promoter; the BRD4 depleted group showed similar results (Figure 6A). We measured the mRNA expression of Notch1 in different groups to validate the accuracy of the ChIP‐qPCR results (Figure 6B). These researches put forward a mechanistic interpretation for BRD4's control of Notch1 expression (Figure 7).

To understand the role of BRD4 in GIC, we mapped the genome‐wide distribution of BRD4 from U251 GIC using CHIP. MACSV1.4.2 (Model‐BasedAnalysis of CHIP Sequence) software uses the drawn readings for peak detection. By comparing IP with input or with the Poisson background model (cut‐off *P* = .05), a statistically significant CHIP concentration area (peak) was identified. Interestingly, the Notch1 region in the original signal map can be visualized directly in a genome browser. We found results consistent with the CHIP‐qPCR: BRD4 was significantly enriched in the Notch1 promoter region (Figure 6D). In GICs, BRD4 peaks were assigned to 21716 genes and localized in intergenic regions (47.71%), intronic regions (31.54%), upstream regions (15.71%), promoters (4.06%), and exons (1.51%) (Figure 6C). Gene ontology (GO) analysis of genes displaying increased BRD4 recruitment in GICs revealed enrichment in biogenesis and RNA metabolic processes (Figure 6E). In addition, we performed a pathway analysis, which is a functional analysis of pathways that map genes to Kyoto Encyclopedia of Genes and Genomes (KEGG) pathways. KEGG pathway test pointed out that significant pathways of cell apoptosis and sugar metabolism were correlated with these screened genes (Figure 6F). These results will provide clues for subsequent research on BRD4 in GICs.

### 3.7 I‐BET151 inhibited tumorigenicity of GIC in intracranial tumor model

In vivo, tumor volume is often used to assess the tumorigenicity of GIC. To explore the potential effect of I‐BET151 on the tumorigenicity of GIC, an intracranial orthotopic xenotransplantation model was established by transplacing U87‐MG GICs into the brain of mice. Intraperitoneal injection of DMSO or I‐BET151 was administered 3 days a week, starting 7 days after implantation, for 3 weeks. We found that the tumorigenicity of GIC was lower in the I‐BET151 group than in the DMSO group by Bioluminescence imaging and hematoxylin eosin staining (Figure 8A, B and D). Mice treated with I‐BET151 survived significantly longer than mice treated with DMSO (*P* < .01) (Figure 8C). In addition, IHC analysis showed that Notch1, Hes1, Ki‐67, CD133, and nestin expressions were decreased in the I‐BET151 treatment group, which showed no difference with the results in vitro (Figure 8E). These results suggest that I‐BET151 inhibits GICs’ tumorigenicity in vivo.

## 4 DISCUSSION

In this study, we identified a new BRD4/Notch1 signaling axis in GICs. We showed that BRD4 modulates the expression of Notch1 and Notch1 signaling pathway activation to regulate self‐renewal and tumorigenicity of GIC. Interestingly, Notch1 is a BRD4‐specific gene because BRD4 interacts with the Notch1 promoter region. These discoveries reveal a new strategy to intervene in GBM recurrence by targeting the BRD4/Notch1 axis of GICs.

The BET family consists of three members that are commonly expressed in mammals: BRD2/BRD3/BRD4, which have two tandem bromine domains at their nitrogen terminals, bind to acetylated lysine residues in histone H3 and H4.^42^ After binding to acetylated chromatin, these proteins regulate transcription by recruiting nucleosome remodeling complexes, chromatin modifiers, and transcriptional co‐activators.^18^ Although their high homology, BET protein does not show the same biological functions between them. Based on TCGA and CGGA databases, we hope to find appropriate therapeutic clues for glioma via simple bioinformatics analysis of BET proteins. In both databases, BRD4 mRNA is inversely associated with OS in glioma patients. Although the OS differences between the groups with high and low expression of BRD2/BRD3 mRNA were significant, the BRD2/BRD3 high group showed a favorable prognosis. Therefore, it seems that BRD2/BRD3 has no value in targeted glioma therapy. Further, we analyzed the relationship between BRD4 mRNA expression and glioma histology and GBM subtype. We found that the BRD4 expression level was highest in GBM, which confirmed the importance of our study on BRD4 in GBM. Significantly, BRD4 is almost not expressed in normal tissue, allowing us to design targeted drugs for BRD4 without worrying about side‐effects in normal tissue. Furthermore, our findings declared that BRD4 expression was relatively high in proneural and classical subtypes. Interestingly, in the classical isoforms, stem cell marker and neural precursor NES together with Sonic hedgehog (SMO/GAS1/GLI2) signaling pathways and Notch (NOTCH3/JAG1/LFNG) were highly expressed. GO classification of the proneural subtype contained developmental processes, cell cycle, and proliferation signatures.^43^, ^44^ Therefore, GICs were suitable for our research.

GICs play a significant biological role in cell populations because of the important characteristics of self‐renewal, sustained tumor growth in vivo, and differentiate into different types of tumor cells.^45^ GICs contribute to GBM's resistance to radiation and chemotherapy, dissemination, and recurrence.^46^ Targeting GIC population may prolong patient survival, but unique weaknesses need to be explored. We demonstrated that I‐BET151effectively inhibited the self‐renewal and proliferation of GICs. Although specific inhibitors of BET proteins are serviceable here, it is significant to pay attention to these compounds, including JQ1, OTX015, and PLX51107, do not distinguish between different BET proteins.^23^, ^47^, ^48^ Therefore, BRD2, BRD3, and BRD4 were separately studied to determine which one among the several of them participate in the management of the self‐renewal of GICs. We confirmed a diminution in the self‐renewal of GIC by showing that expression of Nestin, CD133, and SOX2, was attenuated by I‐BET151 and BRD4 depletion. Our findings buttress previous studies showing that BRD4 modulated the self‐renewal ability and pluripotency in ESCs.^34^, ^49^ It is worth noting that BRD2 affects GIC proliferation. Similarly, several studies have revealed that BRD2 binds to chromatin remodeling complexes and manages transcription processes related to cell proliferation.^50^, ^51^

Further, our study suggests that targeting BRD4 can restrain Notch pathway activation. The Notch1 pathway has been revealed to sustain the stemness phenotype in glioma,^52^, ^53^ which is consistent with our previous research.^32^ Our results confirm that Notch1 is a major link to management of GIC self‐renewal by BRD4. The results of correlation analysis between BRD4 and Notch1 pathway‐related genes and stem cell marker‐related genes also provide a strong basis for this conclusion. Furthermore, we explained that BRD4 regulation of the Notch1 pathway is due to the association between BRD4 and the Notch1 receptor promoter region. Similar to breast cancer and T‐cell acute lymphoblastic leukemia, the regulation of the Notch1 pathway by targeting BRD4 suggests an epigenetic mechanism.^30^, ^54^ It is worth noting other recent findings explained that BRD4 is associated with the promoter of Jagged1, a type of Notch1 pathway ligand that regulates breast cancer tumor cell dissemination.^30^ Therefore, further studies on GICs are needed to determine the mechanism of BRD4‐mediated Notch1 ligands regulation.

Finally, I‐BET151 abated tumorigenicity of GIC in intracranial xenograft models. I‐BET151 inhibited the progression of GIC, which is in accordance with a classical study revealing that I‐BET151 has good permeability of the blood‐brain barrier.^38^ BET bromodomain‐specific inhibitors, including JQ1, dBET6, and OTX015, prolonged murine survival and reduced tumor incidence in an intracranial model.^55^, ^56^, ^57^ Similarly, previous reports^46^, ^58^ showed that preventing GIC progression is a key element to prolonging survival in an intracranial xenograft model. Thus, the development of therapies targeting GIC should be a major goal to promote the effectiveness of chemotherapy and prolong GBM patients’ survival.

## 5 CONCLUSIONS

In conclusion, our findings put forward a novel glioma treatment strategy. Our findings demonstrate that BRD4 is enriched at the promoter region of Notch1, and that BRD4 inhibition is capable of downregulating self‐renewal and tumorigenicity in GICs. This can be a promising strategy for beating GICs. Thus, studies of molecular targeting and/or signaling pathways that target BRD4 and modulate Notch1 promoter region transcription can be performed from now on as they might facilitate the development of an appropriate approach for treating GBMs.

## AUTHORS’ CONTRIBUTIONS

Designing research studies: Zhennan Tao and Xuetao Li. Conducting experiments: Zhennan Tao, Xuetao Li, and Hao Wang. Acquiring data: Zhennan Tao, Xuetao Li, Guangliang Chen, and Zibin Feng. Analyzing data: Zhennan Tao, Haoran Yin, Guozheng Zhao, and Zhitong Deng. Collecting samples: Hao Wang, Zhitong Deng, Haoran Yin, Guangliang Chen, Chaohui Zhao, Yanyan Li, and Ting Sun. Preparing the manuscript: Zhennan Tao, Xuetao Li, and Hao Wang. The authors read and approved the final manuscript.

## CONFLICT OF INTEREST

The authors declare no conflict of interest.

6

## Supporting information

SUPPORTING INFORMATION

SUPPORTING INFORMATION

SUPPORTING INFORMATION

SUPPORTING INFORMATION

SUPPORTING INFORMATION

SUPPORTING INFORMATION

SUPPORTING INFORMATION

## Data Availability

The data that support the findings of this study are openly available in DOI: 10.1002/ctm2.181.
