# Supplementary figures and images for: BRD4 regulates self‐renewal ability and tumorigenicity of glioma‐initiating cells by enrichment in the Notch1 promoter region
Source: Clin Transl Med. 2020 Oct 4;10(6):e181. doi: 10.1002/ctm2.181 (PMC7533052; doi:10.1002/ctm2.181)

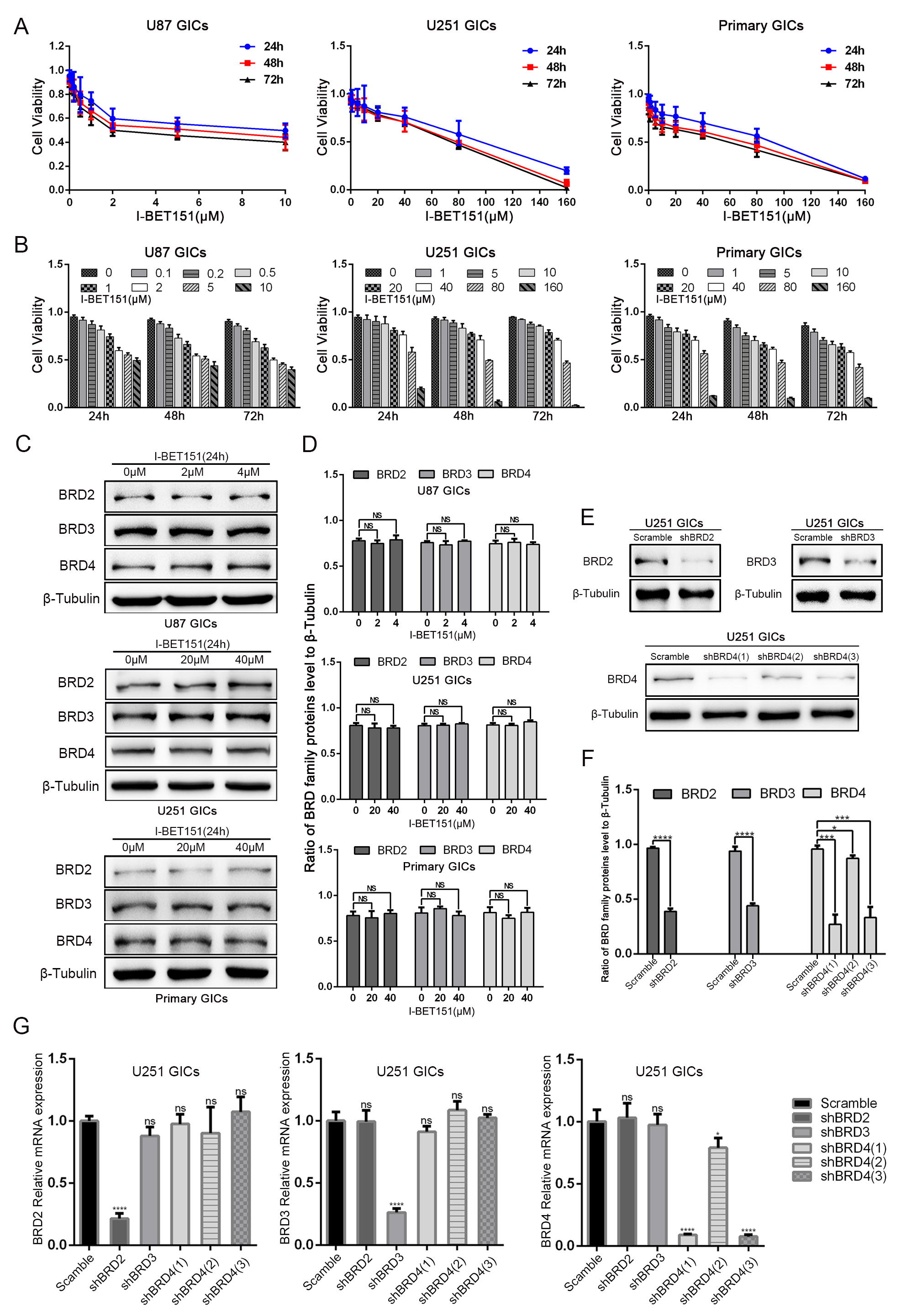

Supplement: Supplementary file 1 — SUPPORTING INFORMATION [file CTM2-10-e181-s001.jpg]

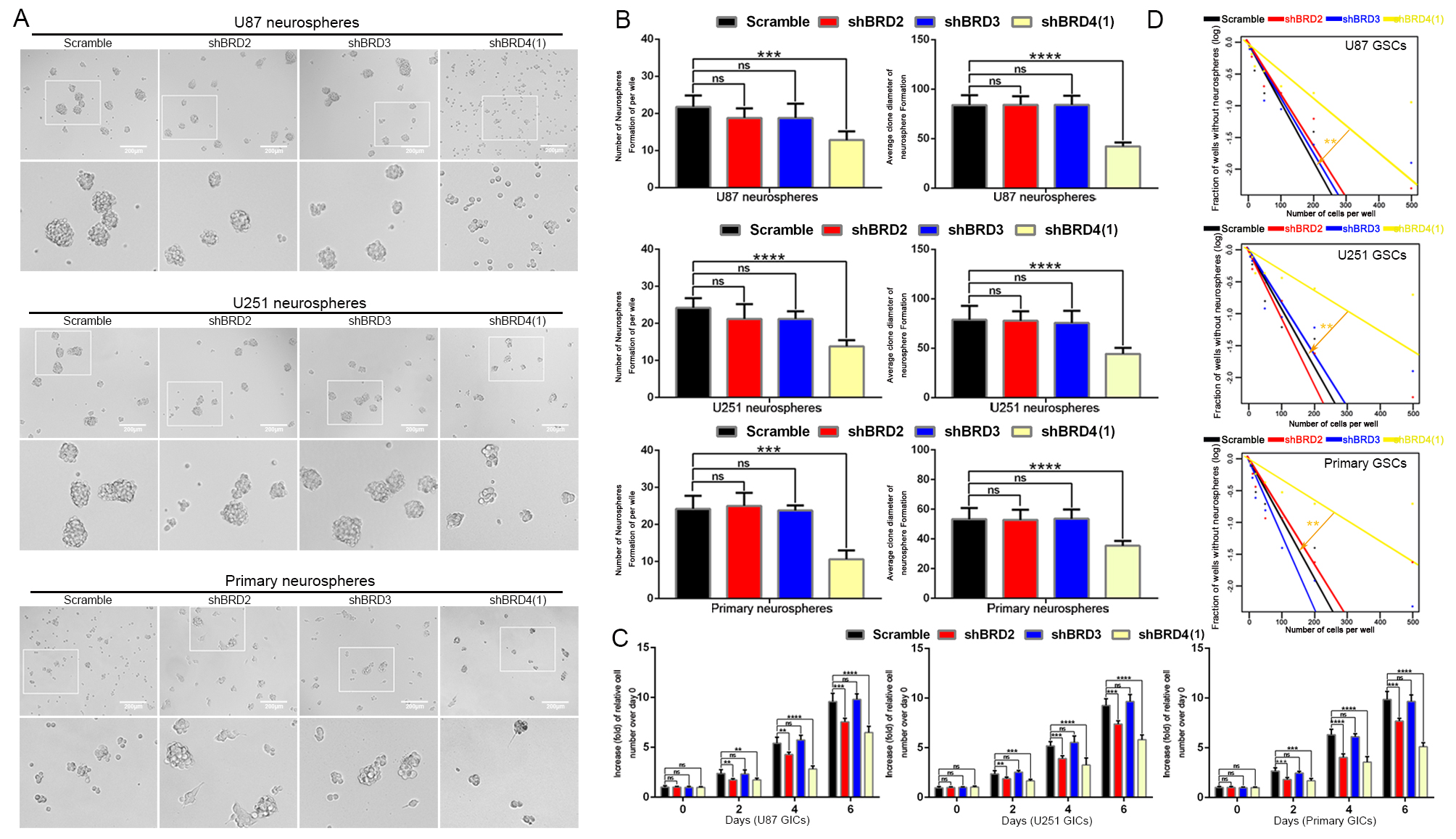

Supplement: Supplementary file 2 — SUPPORTING INFORMATION [file CTM2-10-e181-s002.jpg]

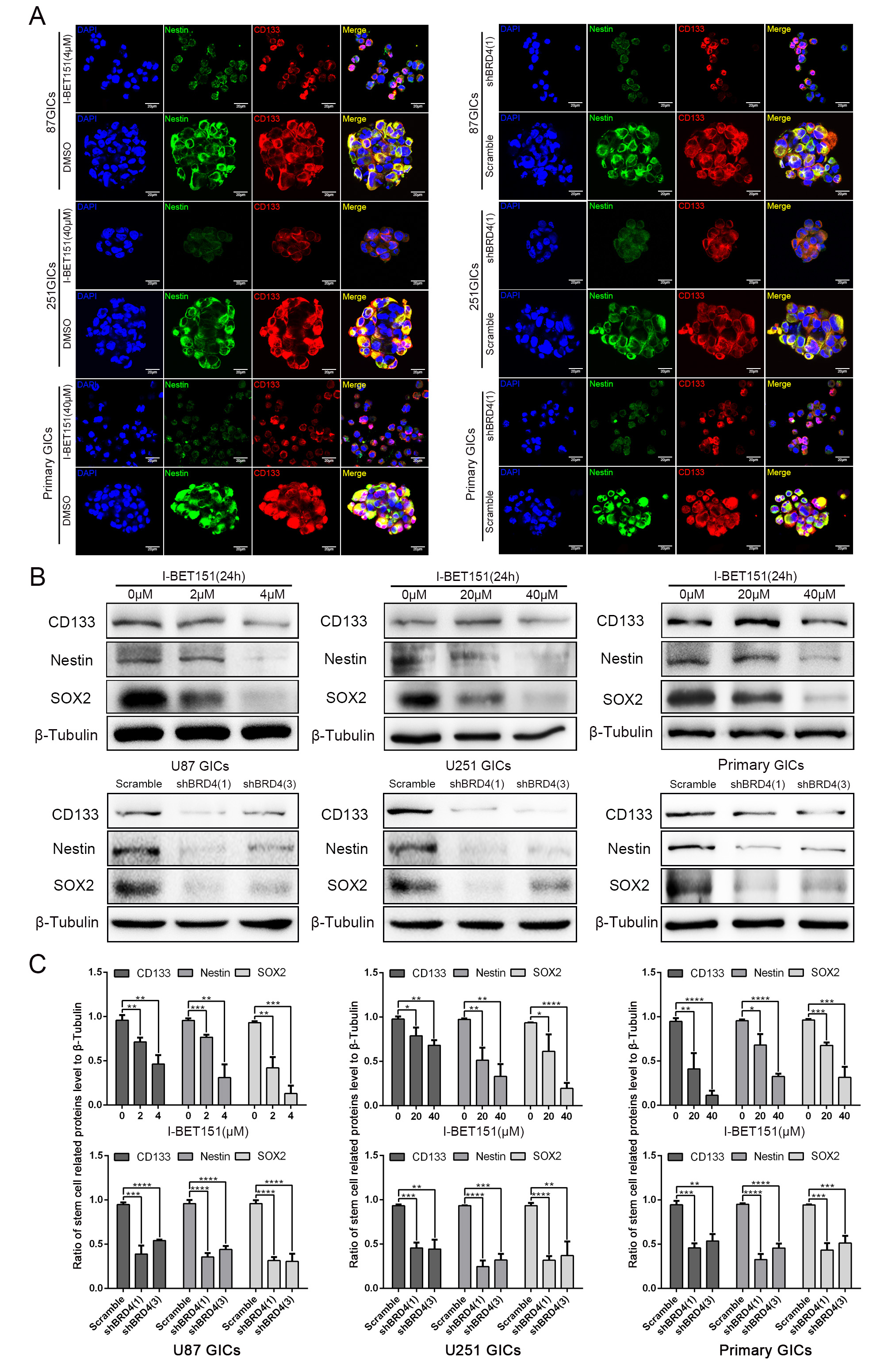

Supplement: Supplementary file 3 — SUPPORTING INFORMATION [file CTM2-10-e181-s003.jpg]

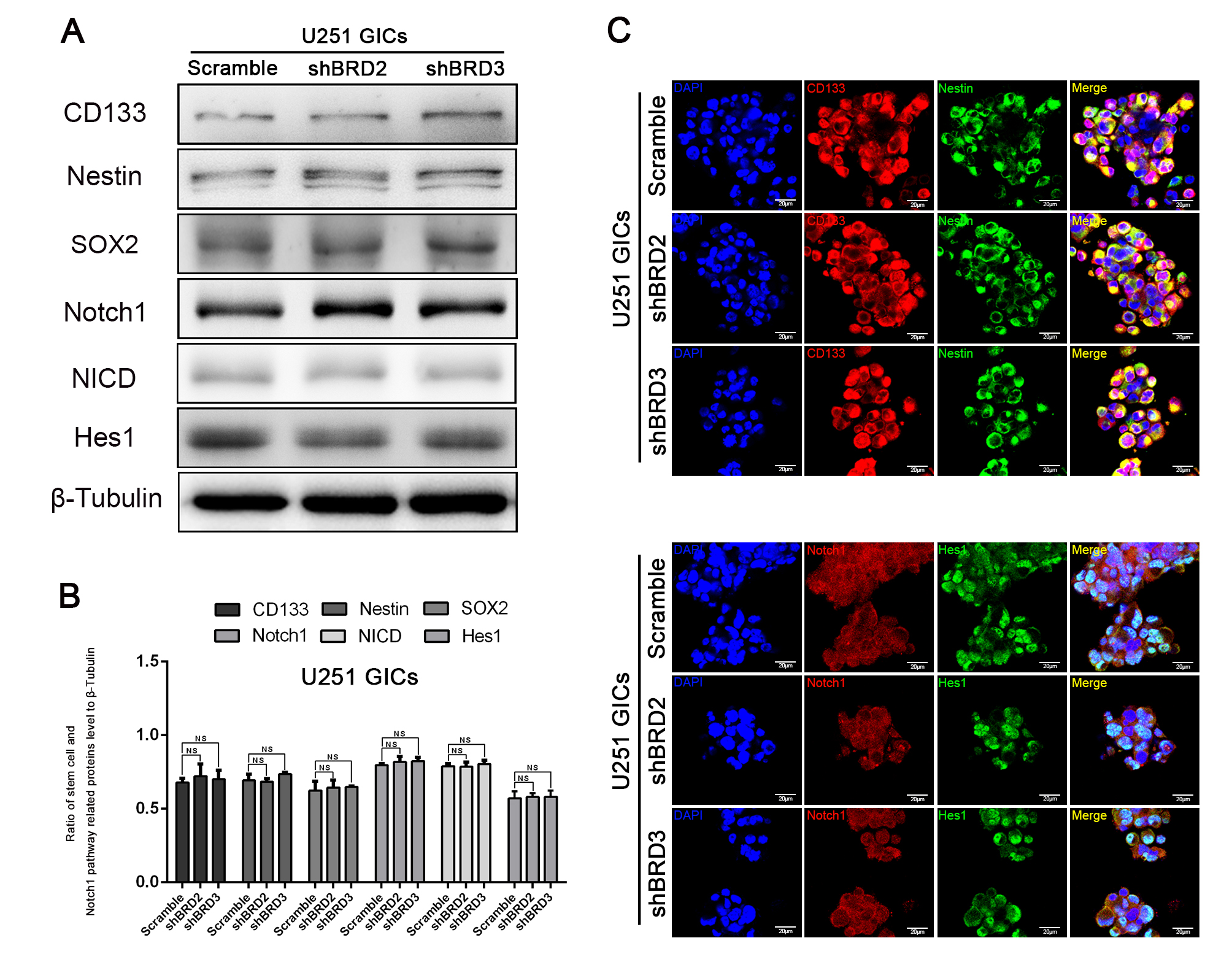

Supplement: Supplementary file 4 — SUPPORTING INFORMATION [file CTM2-10-e181-s004.jpg]

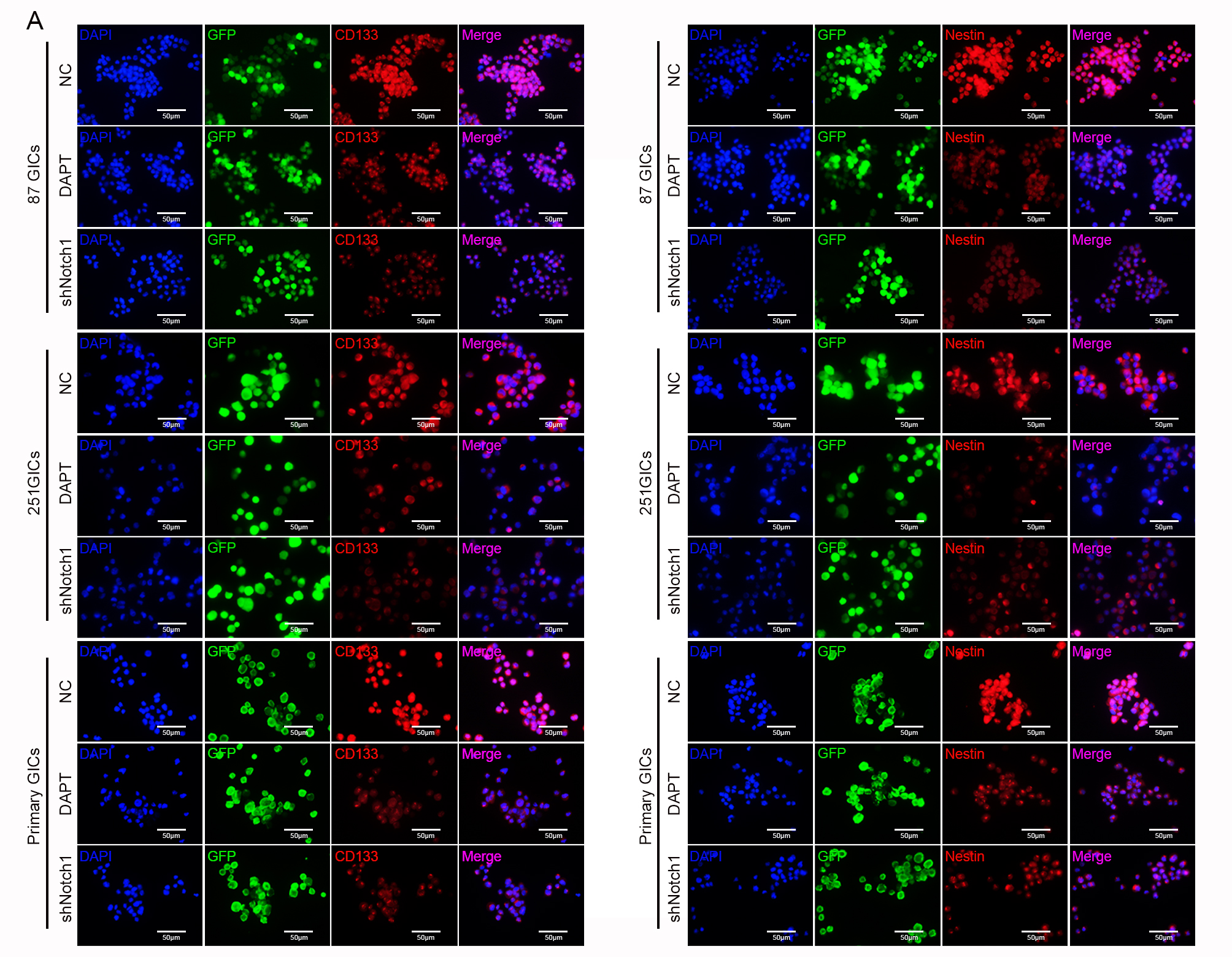

Supplement: Supplementary file 5 — SUPPORTING INFORMATION [file CTM2-10-e181-s005.jpg]

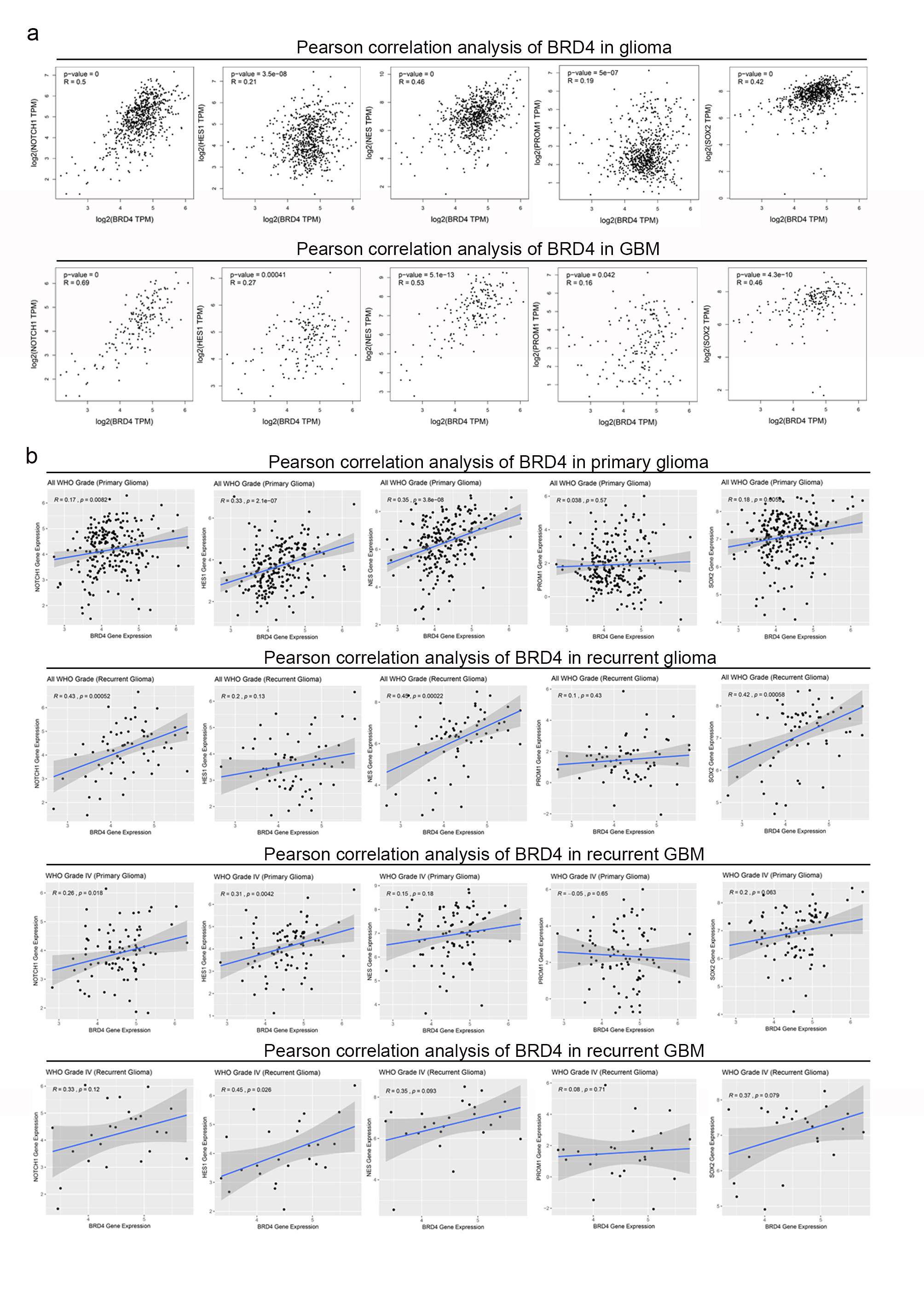

Supplement: Supplementary file 6 — SUPPORTING INFORMATION [file CTM2-10-e181-s006.jpg]
