# Supplementary material for: BRD4 regulates self‐renewal ability and tumorigenicity of glioma‐initiating cells by enrichment in the Notch1 promoter region
Source: Clin Transl Med. 2020 Oct 4;10(6):e181. doi: 10.1002/ctm2.181 (PMC7533052; doi:10.1002/ctm2.181)
Supplement: Supplementary file 7 — SUPPORTING INFORMATION [file CTM2-10-e181-s007.docx]

| Antibody | Catalogue | Producer | Application |
| --- | --- | --- | --- |
| BRD2 | 22236-1-AP | Proteintech | WB,1:1000 |
| BRD3 | 11859-1-AP | Proteintech | WB,1:1000 |
| BRD4 | Ab128874  CST#13440S | Abcam  Cell Signaling | WB,1:1000  CHIP |
| Notch1 | Ab52627 | Abcam | WB, 1:1000  IF, 1:100 |
| NICD | Ab8925 | Abcam | WB, 1:1000 |
| Hes1 | Ab71559  Ab119776 | Abcam  Abcam | WB, 1:1000  IF, 1:100 |
| CD133 | Ab19898 | Abcam | WB, 1:1000  IF, 1:100 |
| SOX2 | CST#3579 | Cell Signaling | WB, 1:1000 |
| Nestin | 66259-1-Ig  Ab105389 | Proteintech  Abcam | WB, 1:1000  IF, 1:100 |
| β-Tubulin | CST#2146S | Cell Signaling | WB, 1:1000 |
| Anti-mouse IgG | CST#7076 | Cell Signaling | WB, 1:2000 |
| Anti-rabbit IgG | CST#7074 | Cell Signaling | WB, 1:2000 |
| Alexa Fluor® Plus 488 | A32723 | Invitrogen | IF,1:500 |
| Alexa Fluor® Plus 594 | A32740 | Invitrogen | IF,1:500 |

**Supplemental Table 1**: Introduction and application of antibody.

**Supplemental Table 2**: shRNAs used in the study.

| Gene | Target Seq (5' to 3') |
| --- | --- |
| BRD2 | CACGAAAGCTACAGGATGT |
| BRD3 | AATTGAACCTGCCGGATTA |
| BRD4(1) | GGAAACCTCAAGCTGAGAA |
| BRD4(2) | GCGTTTCCACGGTACCAAA |
| BRD4(3) | AAACCGAGATCATGATAGT |
| Notch1 | GCATGGTGCCGAACCAATACA |

**Supplemental Table 3**: mRNA expression QPCR primers used in the study.

| Gene | Forward (5’-3’) | Reverse (5’-3’) |
| --- | --- | --- |
| Notch1 | GAGGCGTGGCAGACTATGC | CTTGTACTCCGTCAGCGTGA |
| BRD2 | AATGGCACAAACGCTGGAAAA | CACTGGTAACACTGCCCTG |
| BRD3 | TGCAAGCGAATGTATGCAGGA | CATCTGGGCCACTTTTTGTAGAA |
| BRD4 | GAGCTACCCACAGAAGAAACC | GAGTCGATGCTTGAGTTGTGTT |
| GAPDH | CAGGAGGCATTGCTGATGAT | GAAGGCTGGGGCTCATTT |
